# Detectability of biosignatures in a low-biomass simulation of martian sediments

**DOI:** 10.1038/s41598-019-46239-z

**Published:** 2019-07-04

**Authors:** Adam H. Stevens, Alison McDonald, Coen de Koning, Andreas Riedo, Louisa J. Preston, Pascale Ehrenfreund, Peter Wurz, Charles S. Cockell

**Affiliations:** 1 UK Centre for Astrobiology, School of Physics and Astronomy, University of Edinburgh, Edinburgh, UK; 2 School of Engineering, Bioimaging Facility, University of Edinburgh, Edinburgh, UK; 3 Sackler Laboratory for Astrophysics, Leiden Observatory, Leiden University, Leiden, The Netherlands; 4 Space Research and Planetary Sciences, Physics Institute, University of Bern, Bern, Switzerland; 5 Dept. of Earth and Planetary Sciences, Birkbeck, University of London, London, UK

**Keywords:** Astrobiology, Astrobiology

## Abstract

Discovery of a remnant habitable environment by the Mars Science Laboratory in the sedimentary record of Gale Crater has reinvigorated the search for evidence of martian life. In this study, we used a simulated martian mudstone material, based on data from Gale Crater, that was inoculated and cultured over several months and then dried and pressed. The simulated mudstone was analysed with a range of techniques to investigate the detectability of biosignatures. Cell counting and DNA extraction showed a diverse but low biomass microbial community that was highly dispersed. Pellets were analysed with bulk Elemental Analysis – Isotope Ratio Mass Spectrometry (EA-IRMS), high-resolution Laser-ablation Ionisation Mass Spectrometry (LIMS), Raman spectroscopy and Fourier Transform InfraRed (FTIR) spectroscopy, which are all techniques of relevance to current and future space missions. Bulk analytical techniques were unable to differentiate between inoculated samples and abiotic controls, despite total levels of organic carbon comparable with that of the martian surface. Raman spectroscopy, FTIR spectroscopy and LIMS, which are high sensitivity techniques that provide chemical information at high spatial resolution, retrieved presumptive biosignatures but these remained ambiguous and the sedimentary matrix presented challenges for all techniques. This suggests challenges for detecting definitive evidence for life, both in the simulated lacustrine environment via standard microbiological techniques and in the simulated mudstone via analytical techniques with relevance to robotic missions. Our study suggests that multiple co-incident high-sensitivity techniques that can scan the same micrometre-scale spots are required to unambiguously detect biosignatures, but the spatial coverage of these techniques needs to be high enough not to miss individual cellular-scale structures in the matrix.

## Introduction

The discovery of an ancient lacustrine environment in Gale Crater by the Mars Science Laboratory (MSL) has implications for the history of habitability on Mars. While the presence of liquid water on the planet’s surface in the past has been demonstrated by previous missions through observations of fluvial, glacial and lacustrine landforms^1–4^, MSL’s in-depth investigations of Gale crater’s sedimentary record presents clear evidence of a habitable environment^5^. With MSL’s detailed geochemical history of Gale crater fulfilling its objective to identify habitable environments on Mars, the next step is to search for evidence that these habitable environments were once inhabited, which is an aim of both the ESA ExoMars Rover and the NASA 2020 Rover missions.

Current martian surface conditions are inhospitable to life, but it has been proposed that the Yellowknife Bay sedimentary system was habitable in Mars’ past^5^. This raises the question of whether life could be detected in such an environment now, if it was inhabited in the past. Similar environments, which are widespread on Mars, have been identified as a key target for future astrobiology missions due to the favourable conditions for preservation^6^.

However, any putative martian lacustrine ecosystem would not *a priori* have been directly comparable to similar systems on Earth. Even with the optimistic assumption that life was also present on Mars in the past, we have no reason to assume this martian life would have accumulated a significant biomass. If we avoid the assumption that a martian lacustrine biome would be microbiologically similar to its terrestrial equivalent, this raises the question of whether it would be detectable at all.

Robotic investigations of Mars have so far found no definitive biosignatures. Organic carbon observed today at the surface of Mars is at levels consistent with meteoritic input (300–1200 ppm)^7,8^, and far below the levels observed in terrestrial soils or lacustrine sediments (~0.5–24 wt%)^9^. Recent measurements have shown orders of magnitude of variation in total organic carbon in the stratigraphic column of Gale crater^10^, suggesting deposition of sediments in episodes with varying levels of environmental carbon. Astrobiology missions to date, from the Viking landers to the Curiosity rover, have relied generally on bulk analysis techniques, but near-future missions will carry instruments designed to measure biosignatures at micrometre scales^11,12^. Typically, these life detection techniques aim to characterise organic molecules using methods such as mass spectrometry or infrared or Raman spectroscopy. However, despite their favourable conditions for habitability and potential for preserving biosignatures, sedimentary environments also present challenges for the detection of organic molecules when using these techniques^13^. That the biogenicity of some of the oldest known fossils on Earth remains ambiguous^14^ also brings into question how confidently we might be able to identify biological systems of similar ages on Mars.

Our approach to investigating the martian sedimentary environment was to simulate it in the laboratory using the detailed knowledge of the geochemistry of martian surface environments provided by recent missions. We describe experiments designed to simulate a comparable early martian lacustrine environment using *in situ* data collected by MSL to create an analogue of a Gale crater mudstone that was inoculated with a terrestrial lacustrine microbial community. This inoculated analogue material was then pressed to simulate burial and analysed with a range of standard microbiological assays and biosignature detection techniques, providing insight into how to search for biosignatures in similar martian environments to determine if they were ever inhabited. By using instruments analogous to those carried by the ExoMars and 2020 rovers (bulk mass spectrometry and Raman and infrared spectroscopy) and new instruments in development (Laser ablation Ionisation Mass Spectrometry, LIMS), we can determine if these biosignatures can be detected in similar environments that would be explored by these rovers in the near future.

## Results

### Cell counts and quantitation

**Figure 1:**
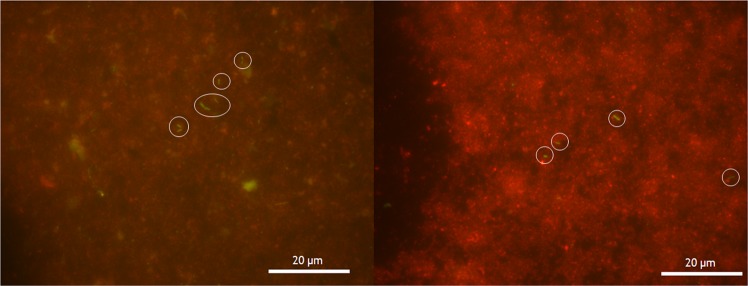
Typical micrographs of stained cells in Mars analogue mudrock. Example micrographs of final transfer cultures. Rod-shaped cell structures, distinctive from spherical structure that may or may not be cellular, are marked with white circles. There were high levels of autofluorescing material (red) of different grain sizes in almost all fields of view.

Cell counts confirmed the presence of microbes in the final of the three transfers of our inoculated mudstone analogue (Fig. 1). The high dilution factor required to reduce the impact of autofluorescing sediment meant that there were generally very few countable cells, with most fields of view containing no cells. Once corrected for the full sample volume, these counts gave a wide range of total cell number in each sample of between 10^5^–107 cells ml^−1^, which is consistent with concentrations considered low – normal biomass for terrestrial soils^15,16^. Given our later analysis, which suggest total biomass far below terrestrial soils, we assume that these numbers are overestimates caused by the effect of small number statistics multiplied by the dilution factors, especially since the detection limit at these dilutions would be 10^4^ cells ml^−1^. The high levels of background fluorescence and autofluorescing material made identifying cells difficult.

### DNA extraction

DNA was successfully extracted from samples of the initial inoculate and the first and second transfers of inoculated sample material. Not all of the final transfer samples had DNA extraction attempted, as the extraction procedure destroys the sample. For those final transfers that did have DNA extraction attempted, no amplifiable DNA was recovered.

**Figure 2:**
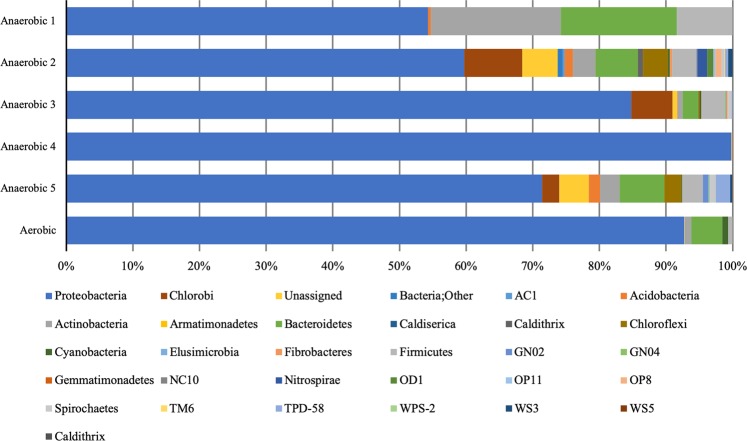
Microbial community composition of transfer samples. Relative abundance of bacterial Phyla in the second transfers of our samples where DNA was recoverable. Phyla with abundance below 1% are not shown. Proteobacteria were dominant in all the samples.

The original inoculate material is a mixed community dominated by Proteobacteria and methanogenic archaea, with a variety of other common marine/sedimentary organisms at lower abundance. Following the inoculation of the first and second transfer, the microbial community in each sample appears to have enriched stochastically, with Proteobacteria remaining dominant in all samples (Fig. 2), but a high degree of variability between samples (Supplementary Fig. 1, Supplementary Table 1). Archaea, while present in the original inoculate, were almost completely absent in the later transfers, but there were no other obvious patterns in the development of the microbial community. Some notable components of the community that showed enrichment or maintenance were *Chlorobiaceae* and *Geobacter*, which were at below 1% relative abundance in the initial inoculate but enriched up to 50% in later transfers. Other Proteobacteria such as *Comamonadaceae*, *Aeromonadacea*, *Oxalobacteraceae* and *Rhodocyclaceae* were also present in increased proportion after multiple transfers.

### Mass spectrometry

#### Bulk EA-IRMS analysis

**Figure 3:**
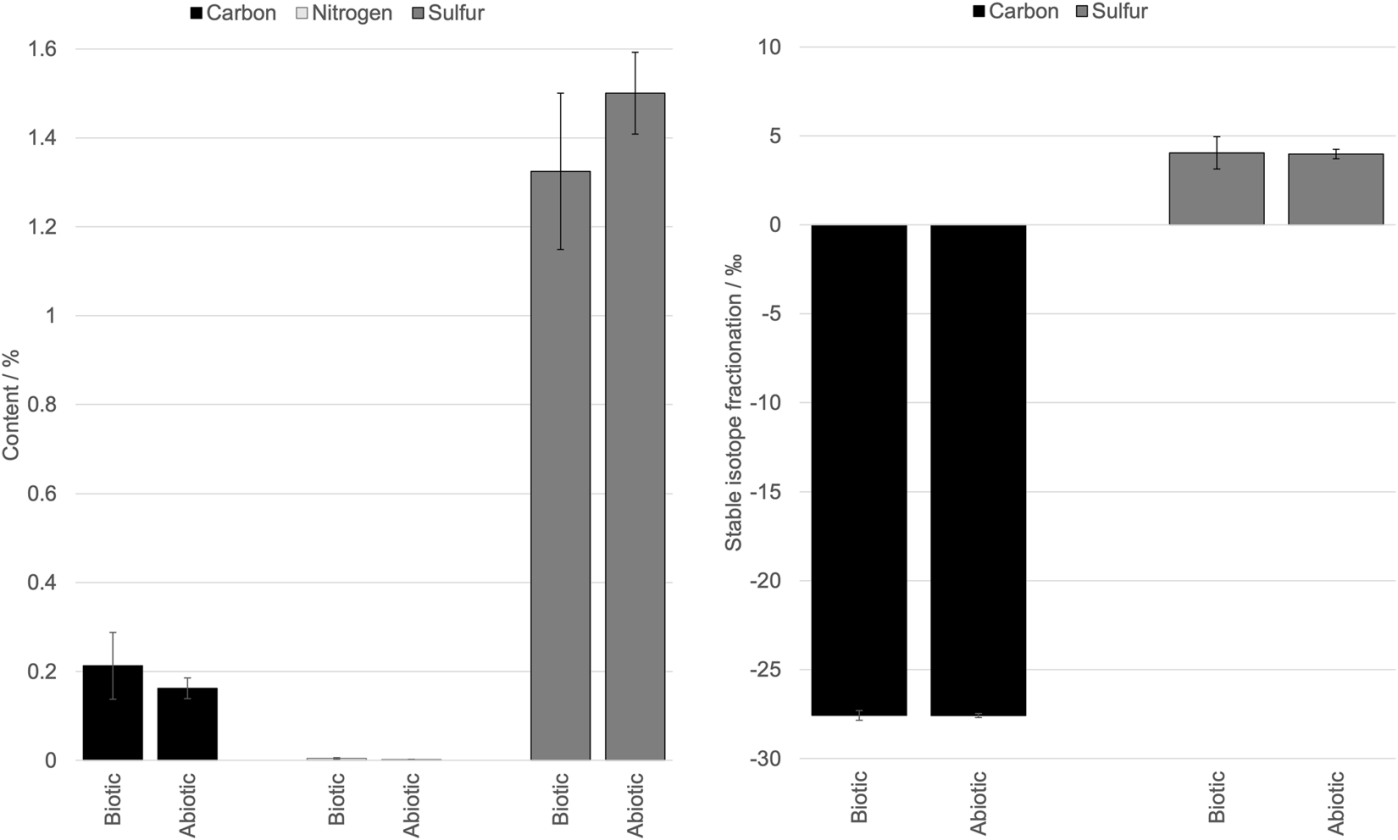
EA-IRMS analysis of bulk composition of samples. Comparison of carbon, nitrogen and sulfur content and stable isotopic fractionation of inoculated samples and abiotic controls from EA-IRMS bulk measurements. There was insufficient nitrogen to measure its isotopic fractionation. Error bars are the standard deviation between multiple analyses of each set of samples.

Bulk EA-IRMS analysis of carbon, nitrogen and sulfur showed minor differences in total content and isotopic fractionation between inoculated and abiotic controls (Fig. 3), at levels below the measurement errors. There was insufficient nitrogen to permit nitrogen isotope analysis. The contribution of the analogue material before inoculation, including each individual mineral in the analogue is shown in Supplementary Fig. 2. There was no measurable difference in total organic carbon or sulfur content or carbon fractionation between unirradiated and irradiated samples (Supplementary Fig. 3). There was a difference in the sulfur fractionation of the unirradiated and irradiated samples, although the difference was small (approximately 3‰) and could be caused by inhomogeneity in the amount of sulfur minerals in each of the samples.

#### LIMS analysis

**Figure 4:**
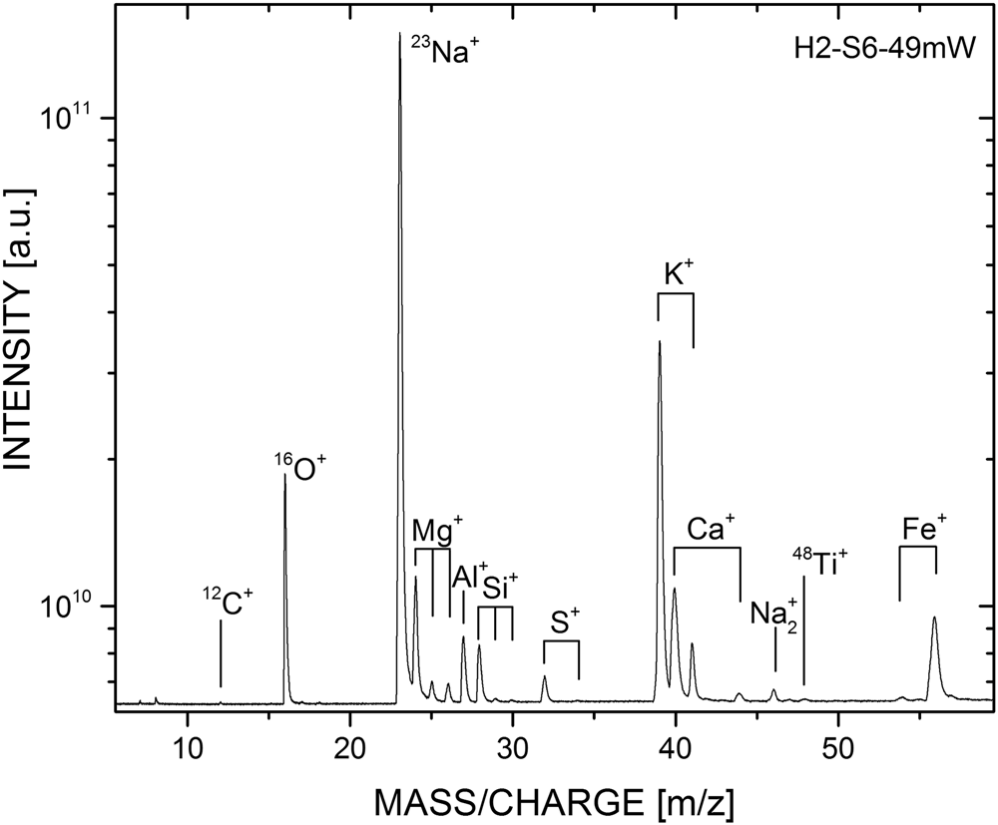
LIMS produced mass spectrum. Representative mass spectrum of the mineral matrix (laser power 49 mW). Major and minor elements are labelled. Here, an accumulation of 71 files are shown, each consisting of 500 single shot spectra.

Due to micrometre scale inhomogeneity of the samples or variable ablation efficiency, spectrum to spectrum variations were expected for LIMS data, both between different spots and at different depths. Averaging of a number of mass spectra yields a representative ‘matrix-background’ spectrum of the host material (e.g. Fig. 4), while simultaneously enhancing signal intensity and SNR.

**Figure 5:**
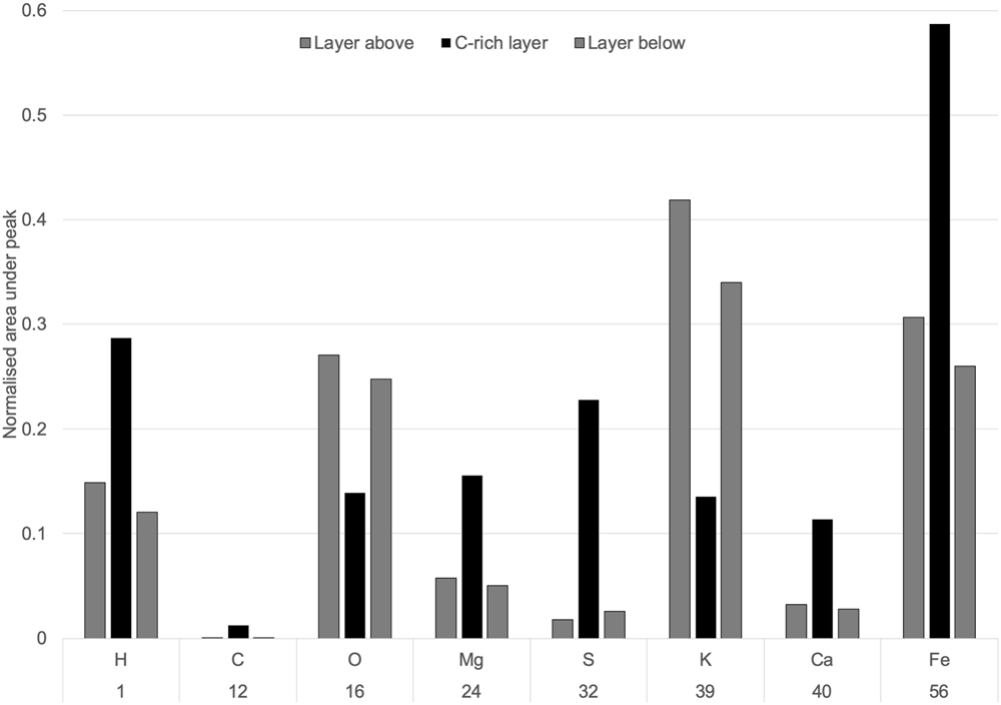
LIMS analysis of sample showing elemental composition changes across vertical layers in locations associated with carbon-enrichment. Integrated area under element peaks from a series of measurements through a carbon rich location in an inoculated sample, normalised to the signal for sodium. There is a clear pattern of enrichment in some elements and depletion in others at locations where elevated carbon values were observed.

Comparing mass spectra between spatial locations (both across the surface of the sample and with depth) allows the identification of enrichments or depletions of particular elements^17^. A number of locations in different samples were identified with an increase in carbon (^12^C) signal. Although from bulk MS measurements we know the mineral matrix carried its own endogenous organic carbon, this carbon should be relatively well distributed within the sample pellets and so these carbon enrichments were interpreted as a biosignature. Eight such spatial enrichments of carbon were identified throughout the measurement campaigns. In these measurements, carbon enrichment was noticeably accompanied by enrichments in hydrogen, sulfur and iron relative to the matrix. The relative enrichment of these elements correlated with carbon was consistent in all of our inoculated samples where such a carbon signal was identified. This pattern is more evident by integrating the signal under each of the elemental peaks (Fig. 5), which highlights other elements that were also enriched or depleted in the same pattern as the carbon enrichment. In contrast, where carbon enrichments were identified in the abiotic control samples, the signal for other elements remained at a consistent intensity, including H, S, and Fe.

### Raman spectroscopy

**Figure 6:**
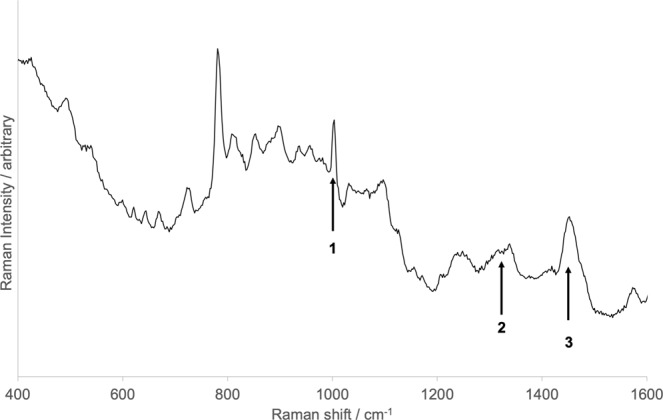
Raman spectrum of Bacillus subtilis. Potential peaks that could be used as identifiers for the organism are marked.

**Figure 7:**
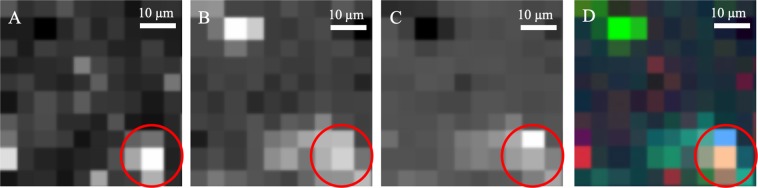
Raman maps in different wavenumber ranges of an analogue sample containing Bacillus subtilis. These wavenumber ranges correspond to the three marked peaks in Fig. 6. (**A**) 996–1034 cm^−1^, (**B**) 1299–1361 cm^−1^, (**C**) 1375–1480 cm^−1^. (**D**) Combined false-colour Raman map of three wavenumber ranges.

Biosignatures were first sought by mapping peaks observed in the control organism (*Bacillus subtilis*). The Raman spectrum of dried *B*. *subtilis* cells on a quartz slide, which matches previously published Raman data for *B*. *subtilis*^18^, is shown in Fig. 6. The area under the peaks of interest was integrated and mapped for an area 50 × 50 pixels, with each pixel representing an area of 2 μm^2^. The maps of the variation in the area of Peaks 1, 2 and 3 from Fig. 6 across the field of view of a *B*. *subtilis* doped sample are shown in Fig. 7. The correlation between bright regions of these maps suggest there is a potentially biological structure in the lower right of the field of view, as this area has multiple Raman peaks associated with *B*. *subtilis*. We can choose any such peak or region to create a map of and combine them to show where there are correlated peaks.

**Figure 8:**
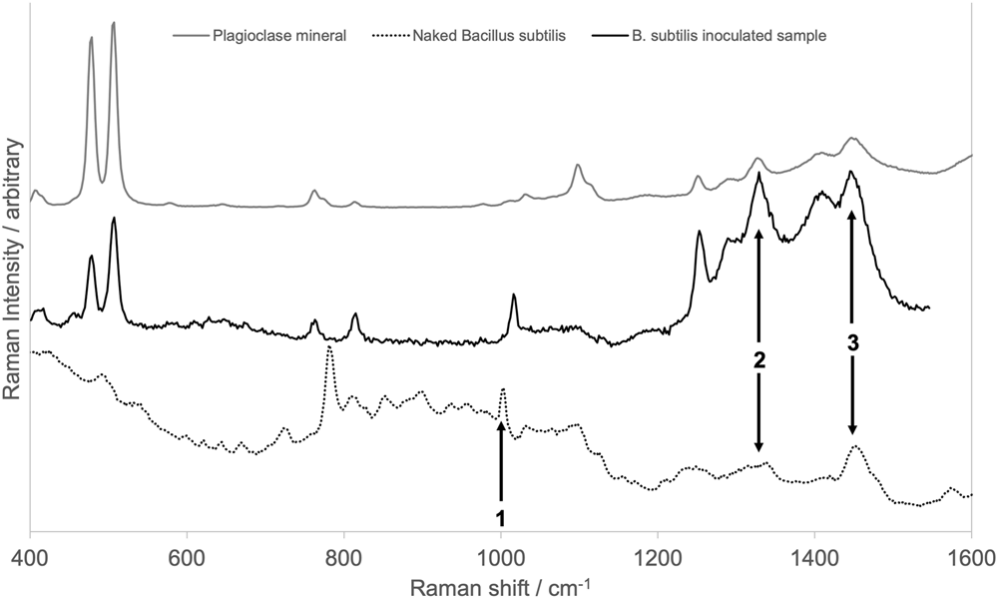
Comparison of Raman spectra from (black) a spot from a Bacillus subtilis inoculated sample (black line), naked Bacillus subtilis cells (dotted line) and the plagioclase mineral used in the analogue material (grey line).

Comparison of the spectrum of the region (or ‘spot’) of interest with the pure *B*. *subtilis* spectrum (Fig. 8) shows that while there are matches for Peaks 1, 2 and 3 (though perhaps slightly shifted), there are other *B*. *subtilis* peaks that are not present in the sample spectrum. A number of peaks from the sample spectrum match a Raman peak of the plagioclase component of the mineral matrix. Because the sample spectrum does not match every peak in either the *B*. *subtilis* spectrum or the plagioclase spectrum completely, this leaves the identification of the material at this location ambiguous – it could be a *B*. *subtilis* cell, a larger plagioclase grain, or a clump of either. Similarly ambiguous results were observed for all Raman maps.

Where a sample contains unknown organisms, as in the environmentally inoculated samples, an initial Raman spectrum for the contained organisms is not available to guide the search for biogenic features. There are many biologically associated Raman features at a wide range of wavenumbers^19,20^. This makes it impossible to derive a general biological Raman spectrum.

**Figure 9:**
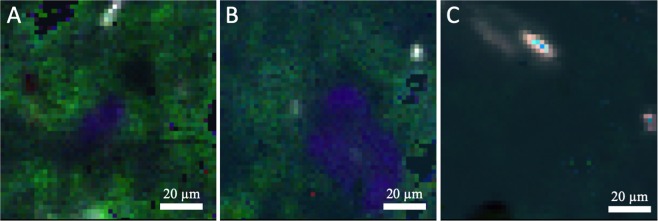
Raman analysis of analogue mudstone samples. Panels A and B are Raman maps of lake-community inoculated sample pellets. Panel C is a Raman map of Bacillus subtilis inoculated sample pellet. Each colour shows the integrated peak area between a minimum and maximum wavenumber. Red = 1375–1469 cm^−1^, Green = 1478–1575 cm^−1^, Blue = 1591–1722 cm^−1^_._

Example maps from environmentally inoculated samples are shown in Fig. 9. Because these were created by manually selecting wavenumber ranges from peaks visible in spectra from across the map, they do not necessarily correspond to biological features. However, there is a spatial correlation in these features that forms structures of a scale of *B*. *subtilis* cells (1–10 µm), and this spatial correlation of the same wavenumber ranges appears in more than one field of view from more than one sample. These wavenumber ranges do not correspond to Raman peaks of any of the minerals in the sample matrix, and are in the same region as a wide range of Raman features of bio-relevant molecules, including lipids, amides, DNA and C/O/H vibrational modes^20^, so we can posit that they are biological features. The noisy background of the maps is likely to be an artefact of peak integration across the high Raman fluorescence that sits in these wavelength ranges.

### FTIR spectroscopy

**Figure 10:**
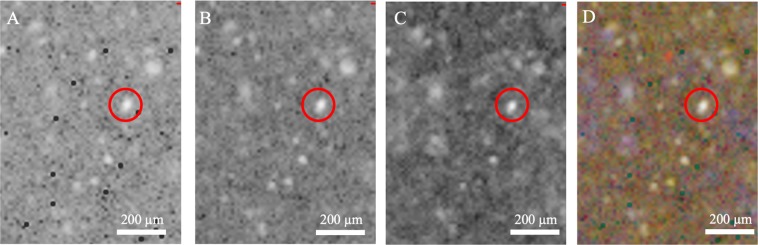
FTIR maps from the same location on an environmentally inoculated sample. Each map shows the intensity of infrared reflection at a distinct wavelength. Panel A shows the 1516 cm^−1^ band associated with the C-C aromatic stretch, panel B shows the 1660 cm^−1^ band associated with the Amide I, panel C shows the 2959 cm^−1^ band associated with the CH_3_ functional group, and panel D shows the combined false colour FTIR map of these three bands.

Similar maps were produced for FTIR scans of the same samples. Figure 10 shows an example set of maps for one sample (the same as Fig. 9A). There are similar sized- and shaped- features as in the Raman maps apparent in some organic-functional-group associated infra-red wavenumber bands. In some cases, these features correlate across multiple organic associated bands, but generally the correlation was less consistent than across the identified biologically associated Raman bands, and some maps showed widely dispersed, unstructured features suggestive of endogenous organic carbon rather than biological structures.

## Discussion

Most analogue studies of Martian environments use materials with known detectable biomass or materials that in some way differ from known Martian environments. In this study, we used an analogue of martian sedimentary materials^13^ to investigate the question of whether this material could in principle support a biota and whether that life could be detected using existing spacecraft instruments.

Our study shows that cell counts were not reliable. Mineral fluorescence from grains with similar sizes to microbes were one complicating factor, but the extremely low biomass confounded attempts to reliably quantify biomass. Another conventional approach to studying biomass is to extract DNA. It was not possible to extract DNA in the third enrichment of our transfer series, despite the presence of cells confirmed by microscopy. This may be attributable to inhibitors in the material, such as clays, or the low biomass, both of which have been previously been shown to limit DNA extraction^21^. The data show that in extremely low biomass Mars-like sedimentary environments, DNA extraction may not be a reliable method to show the presence of biomass. Although cell counts and DNA extractions are associated with extant or recent biomass, our results show that even when biomass is fresh, recovering evidence for it may be challenging. Despite this, in samples where we could extract DNA, the analogue material was shown to host a diverse microbial assemblage including presumptive iron-reducers (*Geobacter*), anoxygenic phototrophs (*Chlorobiaceae*) and heterotrophs (Proteobacteria). This implies that materials on Mars similar to our analogue, including Gale crater sediments, could support diverse microbial taxa at low abundance.

Bulk EA-IRMS analysis of the samples showed no discernible differences in the total content and isotopic fractionation of organic carbon, nitrogen or sulfur between inoculated and abiotic controls, or between un-irradiated and irradiated samples. The major contributors to the total content of all three elements were the mineral substrate and no biological contribution of C, N or S could be detected. The measured isotopic fractionation of carbon was caused by the organic carbon carried by the original environmental mineral samples, with the largest contributor being the saponite fraction, which could not be baked to remove the organic carbon without altering the clay itself. The Y-Mars analogue, and the Yellowknife mudstone it is based upon, is relatively rich in sulfur minerals, especially anhydrite, pyrrhotite and selenite, presenting a mixture of reduced and oxidised sulfur that could be used in biological metabolism but potentially obscuring any changes in sulfur composition that could be indicative of life. The carbon present in the system showed a consistent isotopic fractionation of 𝛿^13^C = −27‰, which is consistent with biogenic carbon, but since this signature was present in the non-inoculated samples we have to assume this was in place in the minerals before the experiments. For sulfur fractionation, the contribution of each of the component minerals was much more variable with strongly positive, strongly negative and neutral fractionation all being present, presumably due to the formation processes of the minerals, but the average bulk fractionation is consistent with mudstones analysed by MSL^22^. These results suggest bulk analysis of sulfur content and isotopic fractionation in martian samples rich in sulfur minerals will be ineffective at identifying biosignatures as even strong fractionation can be ‘swamped’ by a mineral matrix with moderate fractionation. Current and future instruments have the ability to detect individual organic compounds from a bulk sample, which would offer another route for detecting biogenicity, but at these low concentrations this may also be ineffective.

When applied to rock samples, bulk EA-IRMS techniques require the samples to be crushed and prepared in a manner specific to the type of mass spectrometry in use, meaning that the techniques require a minimum sample mass to operate. In environments with low biomass, this means ingesting a high sample mass to retrieve a detectable signal. Because this is chemically and physically destructive of the entire sample mass, it is not an attractive proposition to analyse large amount of rock samples to look for bulk signatures at low biomass, especially where there may be background levels of organic molecules. Organic matter has been detected in martian mudstones at very low levels (2–20 ppm)^10^, but the levels are consistent with meteoritic infall and cannot be assumed to have biogenic origin. Our results demonstrate that in a low biomass environment with background organic matter, bulk MS analysis struggles to detect any signature of life, and the martian context is likely to be even more difficult, given that high molecular mass organic matter that might point more towards a biogenic origin will be degraded by the harsh chemical and radiation environment of the martian surface.

More sensitive mass spectrometry techniques such as LIMS provided qualitative comparisons between samples and overcame some of the disadvantages of bulk analysis. Quantitative analysis would be enabled in space missions by having reference materials available on the spacecraft platform, as in the LAZMA instrument aboard Phobos-Grunt^23^. Nevertheless, our results show that qualitative analysis using this technique can identify spatially constrained biosignatures in Mars analogue environments. Enrichment of carbon alongside multiple bio-relevant elements at micrometre scales appeared to be indicative of the presence of microbial cells. While the presence of carbon alone is not necessarily a biosignature, as it could be present as exogenous organic carbon or in mineral phases such as carbonate, the spatially constrained enrichment of other elements suggests something other than matrix heterogeneity. The fact that an increase in four bio-relevant elements was consistently and exclusively seen in inoculated samples, compared to just enrichment of carbon in abiotic control samples more suggestive of endogenous carbon, strongly indicates that this signature is of biological origin. The small spatial extent of these signals suggests that they are detections of cell-scale structures. The signal is in line with previous studies conducted on micrometre-sized fossil structures, in which biogenicity could be confirmed optically before ablation^17,24^, and could be related to known biological ratios of elements such as the Redfield ratio to add confidence^25^. More confident detection of biogenicity of carbon in these environmental samples would be provided by fine-scale isotopic analysis, as while the bulk isotopic composition may be dominated by exogenous organic carbon input, small scale variations in carbon fractionation would also be a biosignature. LIMS can provide quantitative isotopic data at this scale if appropriate standards are available.

Raman spectroscopy analysis of the samples highlighted the difficulties in using this method to identify life in low biomass Martian sedimentary environments. We found that we could detect peaks putatively linked to biota, but they were not consistent from spot to spot or sample to sample. Even experiments using a defined organism, *Bacillus subtilis*, yielded signatures that were not easily assigned to biota and no sample showed all the peaks that were observed in the purified organism without minerals. Furthermore, some biological peaks lie at the same wavenumber as mineral peaks, so that without prior knowledge of the biota within a sample, it is difficult to confidently assign these to life. These identifications were made more difficult by the high fluorescence background of the material, especially the clay fraction, in the 1000–2000 cm^−1^ range, which further obscured potential biological signals. The Raman spot size may limit the capacity for finding biosignatures. Given the small scale of potential biosignatures observed in our analogue material, a spot spacing associated with the RLS instrument on the ESA ExoMars rover of 100 μm could potentially miss small microbial structures of micrometre size in low biomass samples.

Similar issues were faced with FTIR analysis. While cell-like structures were visible in bands normally associated with particular functional groups of organic molecules, there was little consistency in the presence of these structures in multiple bands, leaving no obvious combined ‘biosignature’ in the analysis. However, because FTIR analysis is more rapid than Raman spectroscopy due to the lower integration times required, the FTIR instrument was able to map much larger areas of the sample surface faster than the Raman instrument. This means that while structures observed in the FTIR maps are even more ambiguously biological than those observed in the Raman maps, appropriate sequencing of wider-scale FTIR analysis to identify potentially interesting locations followed by finer-scale Raman analysis of these locations would be an effective sample triage procedure. This procedure would rely on the ability to track the spatial location of identified structures, or have co-aligned instrument apertures to enable consistent analysis of the same spots.

In fact, the ExoMars instrument suite has exactly the mode of operation in mind. While the RLS instrument has a far lower capability for spatial coverage compared to the Raman instrument used in our analysis, it has a cooperative operation mode where the RLS can be used after identification of potentially interesting targets using the MicrOmega visible/near-IR microscope, which has a wider footprint on the sample. After scanning the sample with MicrOmega, the ExoMars system can move the sample to align with a point identified in the initial analysis and scan using the RLS, followed by laser desorption of exactly the same sample spot for mass spectroscopy in the MOMA instrument^26^. The presence of similar micrometre scale ‘biological’ structures in both our LIMS and Raman data suggests that this operational concept of a wide-field ‘recon’ scan, followed by analysis of the same point by multiple instruments will be critical in being able to provide a more definitive identification of biosignatures in the sample. An instrument that allowed for combined analysis of the same micrometre-scale spot using infrared spectroscopy, Raman spectroscopy, LIMS and potentially other techniques would be a powerful tool for biosignature detection, as when combined these techniques can addresses some of the specific problems faced by each when used on its own^27^.

## Conclusions

In samples cultivated in the laboratory on a Mars-analogue sedimentary rock matrix, a variety of biosignature detection techniques recovered non-definitive detections of biological material. Bulk techniques struggled due to the low levels of biomass in the samples, although compound-specific bulk analysis may reveal information complementary to elemental analysis. High-sensitivity spectroscopic and mass-spectrometric techniques were able to identify biological structures in the samples, but no identification was unambiguous. This implies that multiple spatially synchronised detections using different techniques with high spatial sensitivity would be required for a definitive detection, and this presents an extreme challenge for instrument development. These findings are consistent with other studies, which presented ambiguous biosignatures even in the presence of >20% organic matter^28^. Many suggested biosignatures for future investigations are ambiguous in their biogenicity or not generalisable to all life. Other more generalisable and more easily preserved biosignatures such as biogenic mineral phases^29^ are being targeted by future missions, but our ability to understand how these form and exactly what they look like in low biomass environments is limited by the ubiquitous nature of life on Earth and how deeply we can draw analogies between terrestrial life and putative extra-terrestrial life. The particular challenges of detecting life in low biomass sedimentary systems analogous to martian environments suggests that a key enabling technology will be instruments with multiple coincident detection techniques, and increasing spatial coverage while maintaining spatial and spectral resolution and sensitivity will be a significant challenge for future development.

## Materials and Methods

### Analogue material

The analogue material used was a synthetic analogue produced by mixing commercial minerals to match XRD measurements of a martian mudstone in the Sheepbed unit sampled by MSL. This Y-Mars analogue is a mixture of basaltic alteration products, including major proportions of saponite clay and plagioclase feldspar and minor fractions including a number of sulfur-rich minerals. It is fully described elsewhere^13^.

### Microbial inoculation

Two-gram samples of the Y-Mars analogue were mixed with 4 ml of de-ionised water and inoculated with 100 µl of mud collected from the anaerobic layer at the bottom of Blackford Pond in Edinburgh, UK (55° 55′ 31″N, 3° 11′ 45″W) in 25 ml serum bottles sealed with rubber stoppers *in situ*. Three samples were cultured aerobically and six were cultured anaerobically under a 1 bar 4% CO_2_/96% N_2_ atmosphere. Samples were incubated at room temperature for three months in covered glass serum bottles and then 50 µl of the culture was transferred to new bottles of analogue material and water. Three such transfers were made, for a total culture period of nine months.

Control samples were prepared. Triplicate samples of 2 g Y-Mars material and 4 ml water were mixed with overnight cultures of *Bacillus subtillis* (NCIB 3610). This organism was chosen as it is well characterised and has a well-defined Raman spectrum. The cultures were diluted to cell densities of 10^3^, 10^5^ and 10^7^ cells ml^−1^ respectively, measured via optical density (OD) and plate counts, chosen to approximate cell density in typical low-to-high biomass terrestrial soils^15,16^ and to match the cell counts in the environmentally inoculated samples. These samples provided a controlled level of biomass and included only a single species compared to the more diverse environmental samples.

Negative controls were prepared by either mixing 2 g of analogue material with filter-sterilised deionised water or 2 g of dry analogue material on its own and incubating under the same aerobic and anaerobic atmospheres.

### DNA extraction

DNA was extracted from all of the material from the first and second transfers and three of the final transfer samples (two anaerobic and one aerobic) using the PowerMax® Soil DNA Isolation Kit (MoBio Laboratories Inc., USA) following the manufacturer’s instructions, which includes physical disruption of cells using bead-beating. The extracted DNA was sequenced to determine the microbial community in the samples grown in the Y-Mars analogue material. Extracted genomic DNA was sequenced using the Illumina MiSeq platform (Research and Testing Laboratory of the South Plains, Lubbock, Texas, USA). Initial trimming, denoising and chimera checking were carried out by Research and Testing Laboratory^30–32^. Operational taxonomic unit (OTU) clustering and taxonomic identification were carried out in QIIME^33^. Open reference OTU picking was performed against the Silva database^34^ and the remaining unaligned sequences clustered de novo using UCLUST. Singleton OTUs and OTUs that did not align with PyNAST were removed.

### Cell enumeration

Cell counts were made on a portion of the material in the final transfer cultures. 250 µl samples of the inoculated Y-Mars ‘slurry’ were stained using either 5 µl of SYBR Gold (ThermoFisher Scientific) or DAPI (2-(4-amidinophenyl)−1H -indole-6-carboxamidine) in 1 ml of Phosphate Buffered Saline (PBS), incubated and filtered onto Whatman 0.22 μm Cyclopore Black Membrane Filters. Micrographs were collected using a fluorescent microscope (Leica DM4000B) with either an I3 or DAPI filter cube at 100× magnification. Cells were counted in 100 random fields of view for each sample and extrapolated to give the expected number of cells per millilitre of mixed analogue and water.

### Pelleting

All of the samples not used in the DNA extractions (4 anaerobic and 2 aerobic inoculates, positive *B*. *subtilis* controls, and negative controls) were pressed into pellets to simulate burial and form a simulated mudstone. Given that Gale Crater is around 4.5 km deep^5^ and assuming a mudstone density of around 2200 kg m^−3^ ^35^, the overburden pressure on the sediments forming the Sheepbed mudstone would have been around 35 MPa, assuming an upper limit of the crater being completely filled with sediment. Y-Mars pellets were created by pressing dried samples of the powder in a 13 mm diameter stainless steel press, cleaned with ethanol between each pellet. 35 MPa pressure was supplied by a pneumatic vise.

### Irradiation

Pellets were cut into two pieces and half of each was irradiated with a Cobalt-60 gamma ray source. The samples were supplied with an absorbed dose of 101.5 kGy, which approximates 100 Ma under a dose rate at a burial depth of 3 m of 1.8 mGy yr^−1^, as modelled by Dartnell *et al*.^36^ and measured at the surface by MSL^37^. Though the total dose rate experienced by the equivalent material on Mars is unknown, we can assume that until the Sheepbed mudstone was excavated to a depth of around 10 m, the dose rate would be negligible due to protection from the overlying rock^36^. This total dose therefore represents a practically achievable simulation of what martian mudrock might have experienced after erosion of the units above those measured by MSL.

### Analytical methods

A number of mission relevant techniques were used to analyse the simulated mudstone pellets. Mass Spectrometry (MS) is a standard technique that has been used on many planetary missions, including the Surface Analysis on Mars (SAM) instrument on the MSL rover^38^ and the planned Mars Organic Molecule Analyser (MOMA) on the ExoMars 2020 Rover^39^. Raman spectroscopy is a relatively recent addition to astrobiological missions but is planned to be included on both the ExoMars and NASA 2020 rovers^26,40^. In the ExoMars rover package the Raman Laser Spectrometer (RLS) will be accompanied by a near-infrared microscope, MicrOmega^41^. A miniaturised Laser-Ablation Ionisation Mass Spectrometer (LIMS) currently in development is a novel technique designed for the chemical analysis of planetary materials with high sensitivity and micrometre scale spatial resolution.

### Mass spectrometry

Composition and isotope analysis for carbon, nitrogen and sulfur was performed by Iso-Analytical Ltd., UK. In brief, C, N and S isotope analysis of the acid washed sediment samples was undertaken by Elemental Analysis - Isotope Ratio Mass Spectrometry (EA-IRMS), using a Europa Scientific elemental analyser. For C isotope analysis, samples were acidified with 1 M hydrochloric acid and left overnight to allow inorganic carbon to liberate as CO_2_, then neutralized by washing with distilled water and oven dried at 60 °C. The reference material used during ^13^C and ^15^N analysis was IA-R001 (wheat flour, ^13^C_V-PDB_ = −26.43‰, ^15^N_Air_ = 2.55‰) and for ^34^S analysis was IA-R061 (barium sulfate, ^34^S_V-CDT_ = +20.33‰).

The laser ablation ionisation mass spectrometry (LIMS) instrument^42^ consists of a miniature (160 mm × Ø 60 mm) reflectron-type time-of-flight mass spectrometer and a femtosecond laser system as an ablation/ionization source (λ = 775 nm, pulse repetition rate ≤1 kHz, pulse width τ ~ 190 fs, and pulse energy ≤1mJ). Further details of this non-commercial system are available elsewhere^17,43,44^. The full measurement campaign will be detailed in an accompanying publication^45^. Laser pulses are guided towards the vacuum chamber where the miniature LIMS system is located (base pressure 10^−8^ mbar). A lens system above the mass spectrometer focuses the laser pulses through the mass analyser towards the micro-translation-stage mounted sample holder, where sample pellets were mounted using vacuum compatible copper tape. The optical system allows ablation spot sizes of ~ 10–20 μm on the sample surface and each pulse ablates and partially ionises a layer of sample material. Positively-charged ablated ions enter the ion optical system, where they are accelerated, confined and focused towards the field free drift path of the mass analyser. At the ion mirror the ions are reflected towards the detector system^46^ and their mass-to-charge ratio measured in sequence according to their time-of-flight. A segmented anode for electron capture was used and the electric signals are measured with an ADC (sampling rate 2GS/s, vertical resolution 8 bits). For each laser pulse a time-of-flight spectrum was recorded and converted to a mass spectrum using a quadratic equation in a custom Matlab program^47^.

On each sample a systematic set of measurements was collected by varying the laser irradiance with an applied laser power in the range of 29 to 77 mW. Each measurement was conducted at a fresh sample location with 60,000 laser pulses applied at each location. In line with previous studies, 500 time-of-flight spectra were accumulated on-board the acquisition system and saved for a total of 120 files per measurement location on the sample. Each recorded file represents a layer of material with a thickness of around a few hundred nanometres. This measurement procedure allows a chemical depth profiling of tens of micrometers of the investigated material. The measurement results discussed are of qualitative nature only, but can be used to compare measurements from the same sample matrix at the same instrumental settings. To allow comparison between spectra, they were normalized to the Na peak area, which was a consistent and strong signal in every spectrum collected. It should be noted that in this study the LIMS system was optimized to detect individual elements, but such a system can be operated in a mode more optimized for detection of specific organic compounds^48^.

### Raman spectroscopy

Raman spectra were acquired with a confocal Raman microscope (Renishaw inVia) combined with an upright microscope (Leica DMLM). A 100x/0.9 N.A. objective lens (Leica, HCX PL Fluotar) was used to focus the 785 nm excitation laser on to the surface of the sample. The instrument has a measured spot size of 1.2 µm (lateral) and 4 µm (axial) and a wavelength dependent spectral resolution of between 6.5 cm^−1^ and 7.6 cm^−1^. The scans were acquired with 10 s exposure time and 70 mW excitation power for most materials. No sample damage was observed with the maximum laser power on all samples investigated. Wire 2.0 software was used for data acquisition and for cosmic ray removal from raw spectra. Spectra are presented without background subtraction. Due to the time intensive nature of collecting Raman maps, spectra were collected across reduced wavenumber ranges – either 1050–2100 cm^−1^, to target potential diagnostic peaks identified by Ellery and Wynn-Williams^19^, or 400–1550 cm^−1^ to target lower wavenumber peaks identified for *Bacillus subtilis*. Using these reduced ranges, a 100 × 100 μm map took around 18 hours to collect, so in some cases 50 × 50 μm maps were collected, to reduce the time for each sample to a reasonable amount.

A reference Raman spectrum for *Bacillus subtilis* was collected. Two hundred microlitres of an OD^600^ = 1 *B*. *subtilis* culture was dried onto a quartz slide in a sterile flow hood at 10% relative humidity. A Raman spectrum was acquired with the same laser settings as the mineral samples.

Maps were created using scans collected at 2 or 10 μm spacing by selecting peaks in individual spectra. Each scan was visually inspected for peaks, and a greyscale map created with the value for the area under that peak for each pixel. Multiple greyscale maps were combined into 3-channel false-colour RGB images.

### Fourier-transform infrared spectroscopy

Fourier transform infrared (FTIR) spectroscopy maps were collected in reflection geometry using a Thermo-Scientific Nicolet iN10-MX, equipped with a liquid-nitrogen cooled MCTA detector. Background spectra, collected using reflection from a gold-coated mirror, were subtracted from individual spectra. Data were collected using a 25–40 μm^2^ aperture (controlled by motorised slits) at 16 cm^−1^ (ultrafast) or 4–8 cm^−1^ (regular) spectral resolution in the 675 to 7200 cm^−1^ wavenumber range. Maps consisted of regular grids of up to 12,000 individual unpolarised absorption measurements collected at 12–20 μm spacing, ensuring overlap between adjacent measurement locations. Ultrafast map collections contained one acquisition at each position (0.113 s per point). When analysing the FTIR data we searched the spectra for signs of biologically-associated functional groups such as aliphatic and aromatic hydrocarbons, alkenes, alkynes, nitrogen groups such as amines and amides, and carbonyl groups that include aldehydes, ketones, esters and carboxylic acid^49–52^. FTIR maps show the intensity of a single spectral wavelength of interest as a function of sample position.

## Supplementary information

Supplementary Materials

## Data Availability

Data is available via datashare.is.ed.ac.uk, 10.7488/ds/2575.
