# Supplementary material for: Detectability of biosignatures in a low-biomass simulation of martian sediments
Source: Sci Rep. 2019 Jul 4;9:9706. doi: 10.1038/s41598-019-46239-z (PMC6609699; doi:10.1038/s41598-019-46239-z)
Supplement: Supplementary file 1 — Supplementary Materials [file 41598_2019_46239_MOESM1_ESM.docx]

Detectability of biosignatures in a low-biomass simulation of martian sediments

Adam H. Stevens*^1^, Alison MacDonald^2^, Coen de Koning^3^, Andreas Riedo^3,4^, Louisa J. Preston^5^, Pascale Ehrenfreund^3^, Peter Wurz^4^, Charles S. Cockell^1^

1. UK Centre for Astrobiology, School of Physics and Astronomy, University of Edinburgh, UK
2. Bioimaging Facility, School of Engineering, University of Edinburgh, Edinburgh, UK
3. Sackler Laboratory for Astrophysics, Leiden Observatory, Leiden University, The Netherlands
4. Space Research and Planetary Sciences, Physics Institute, University of Bern, Switzerland
5. Dept. of Earth and Planetary Sciences, Birkbeck, University of London, UK

*adam.stevens@ed.ac.uk

Figure 1 Relative abundance of bacterial Classes in the second transfers of our samples where DNA was recoverable. Classes with abundance below 1% are not shown. There was little similarity between the different samples, although some classes showed enrichment.


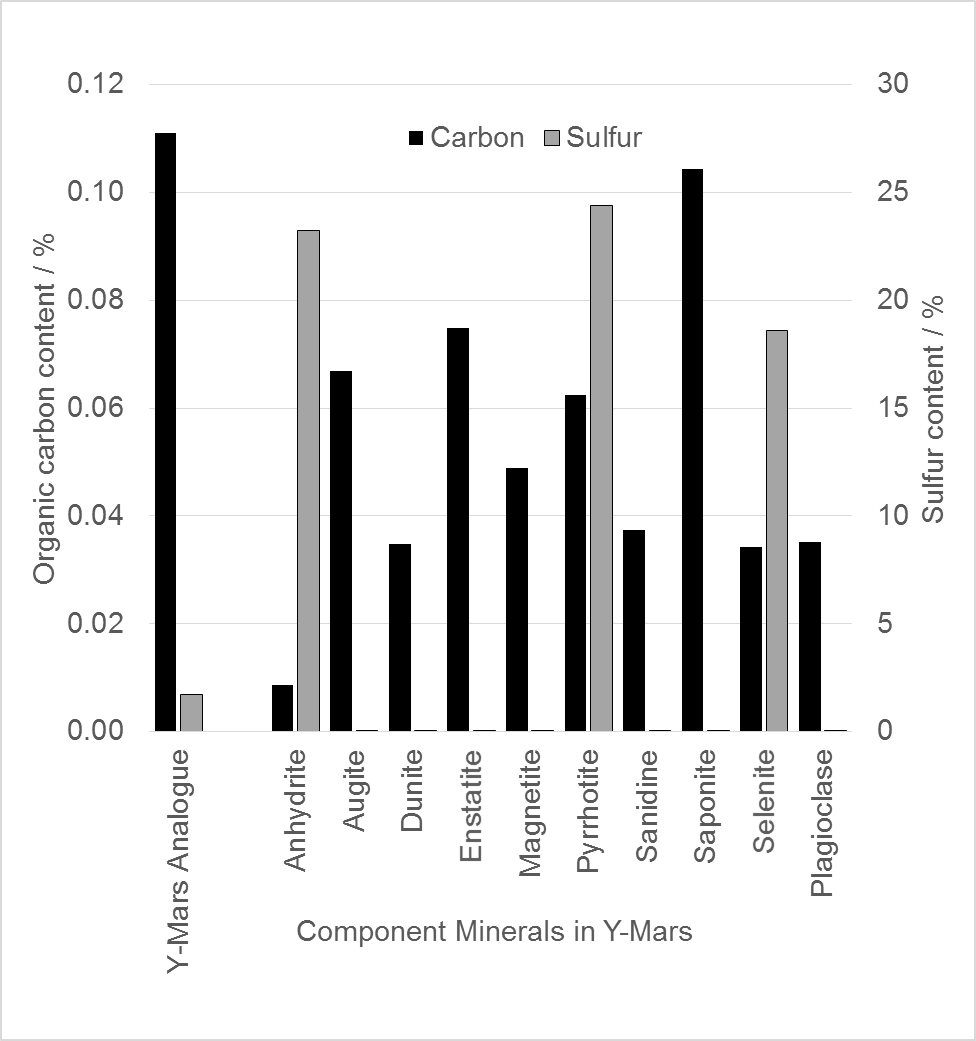

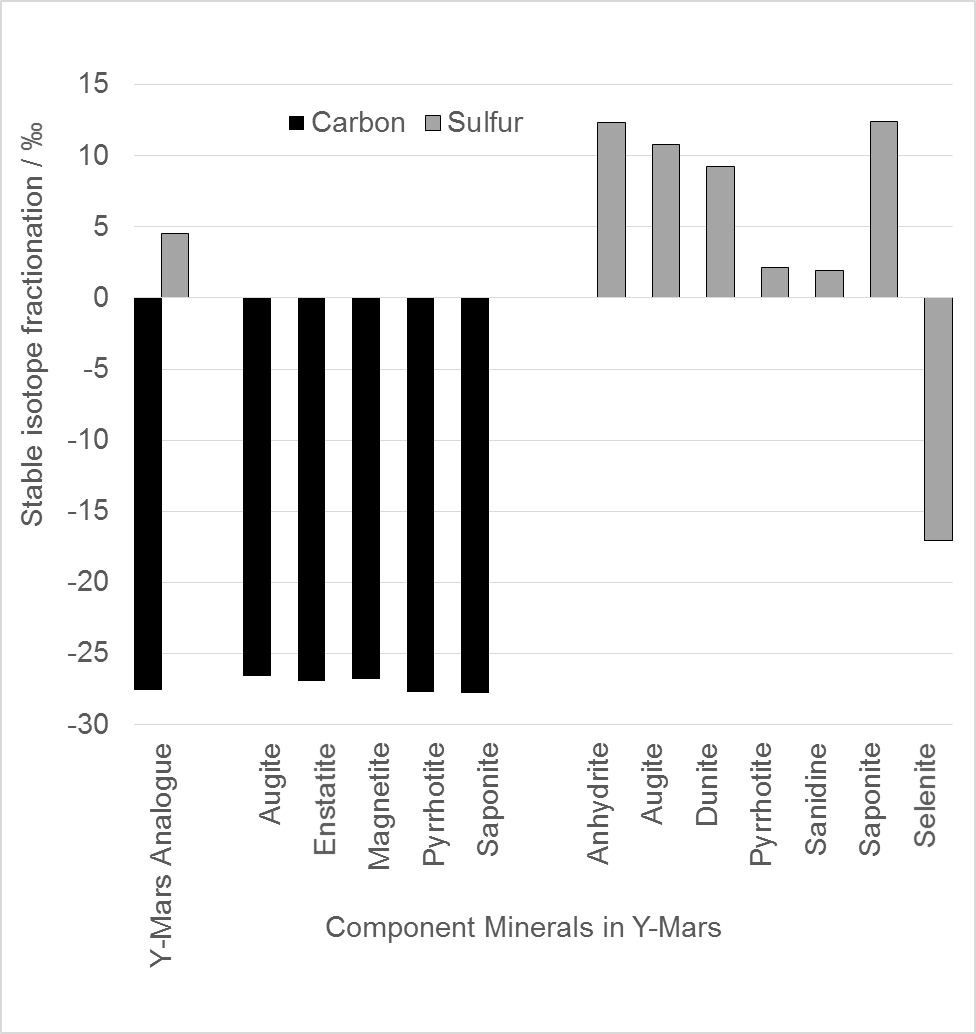


Figure 2 - Comparison of carbon and sulfur content and stable isotopic fractionation of the components of the Y-Mars analogue and the combined analogue material.


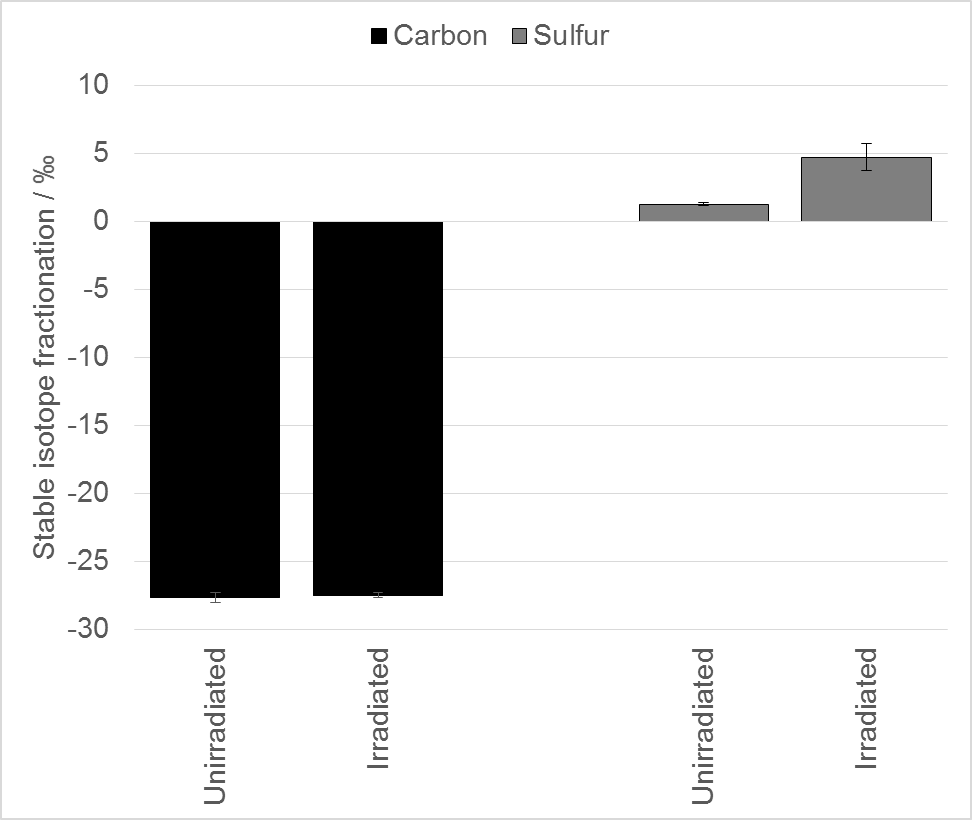

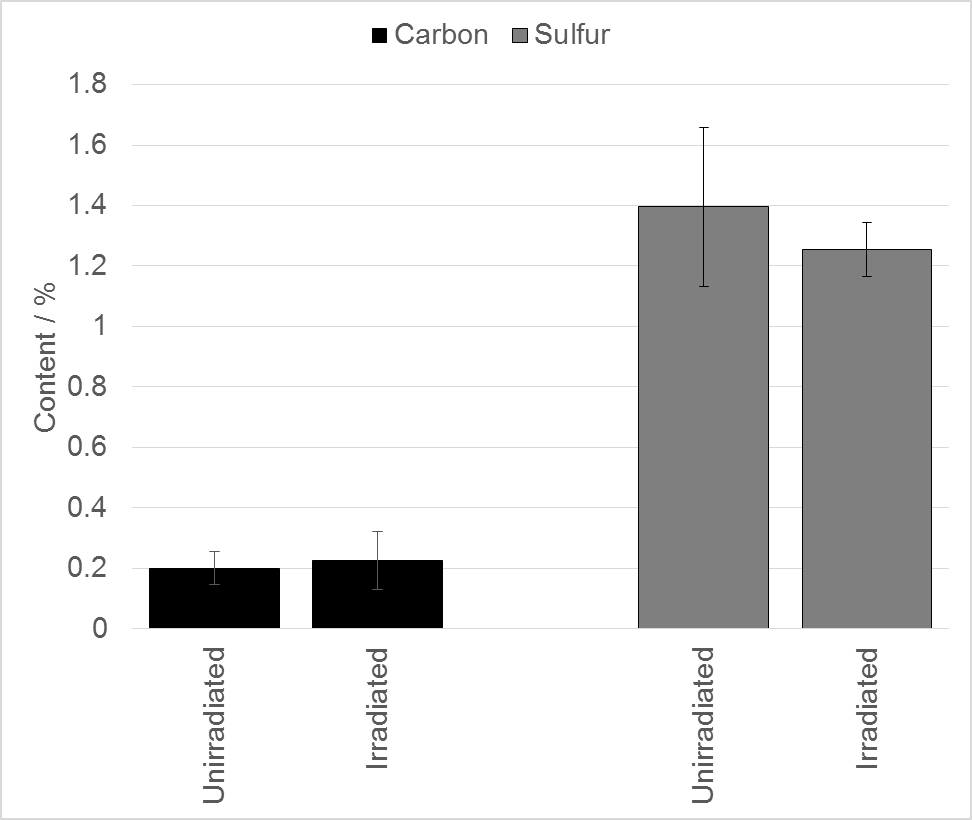


Figure 3 - Comparison of carbon, nitrogen and sulfur content and stable isotope fractionation of inoculated samples with and without an absorbed dose of 101.5 kGy. The abundance of nitrogen was too low to quantify the content or isotope fractionation.

|  | **Aerobic Inoculates** | | | |  | **Anaerobic Inoculates** | | | | | | | | | | | |
| --- | --- | --- | --- | --- | --- | --- | --- | --- | --- | --- | --- | --- | --- | --- | --- | --- | --- |
|  | **Transfer 1** | |  | **Transfer 2** |  | **Transfer 1** | | | | | |  | **Transfer 2** | | | | |
| **#OTU ID** | **AE1** | **AE2** |  | **AE2** |  | **AN1** | **AN2** | **AN3** | **D1** | **D2** | **D3** |  | **AN1** | **AN2** | **D1** | **D2** | **D3** |
| Bacteroidetes;Sphingobacteriia;Sphingobacteriales;Sphingobacteriaceae; |  |  |  |  |  |  |  |  |  |  |  |  |  |  |  |  | 17% |
| Chlorobi;Chlorobia;Chlorobiales;Chlorobiaceae; |  |  |  |  |  | 1% | 3% | 24% | 3% | 36% | 21% |  |  |  | 3% | 2% |  |
| Chlorobi;Chlorobia;Chlorobiales;Chlorobiaceae;Other |  |  |  |  |  |  | 49% | 12% | 1% | 1% | 10% |  |  |  | 2% | 6% |  |
| Firmicutes;Bacilli;Bacillales;Paenibacillaceae;Paenibacillus |  |  |  |  |  |  |  |  |  |  |  |  |  |  |  |  | 7% |
| Proteobacteria;Alphaproteobacteria;Sphingomonadales;Sphingomonadaceae;Sphingomonas |  |  |  |  |  |  |  |  |  |  |  |  |  |  |  |  | 12% |
| Proteobacteria;Betaproteobacteria;Burkholderiales;Oxalobacteraceae;Other | 56% | 40% |  |  |  |  |  | 11% |  |  |  |  | 1% |  |  |  | 11% |
| Proteobacteria;Betaproteobacteria;Burkholderiales;Oxalobacteraceae; |  |  |  |  |  |  |  |  |  |  |  |  |  |  |  |  | 17% |
| Proteobacteria;Betaproteobacteria;Burkholderiales;Comamonadaceae; |  | 6% |  |  |  |  |  |  | 2% |  |  |  |  | 94% | 1% |  | 2% |
| Proteobacteria;Betaproteobacteria;Other;Other;Other |  |  |  |  |  |  |  |  |  |  |  |  |  |  | 11% |  |  |
| Proteobacteria;Betaproteobacteria;Rhodocyclales;Rhodocyclaceae;Other |  | 2% |  |  |  | 2% |  | 3% | 2% | 3% | 3% |  |  |  |  | 1% |  |
| Proteobacteria;Gammaproteobacteria;Aeromonadales;Aeromonadaceae; |  |  |  |  |  |  |  |  |  |  |  |  | 1% | 2% |  |  |  |
| Proteobacteria;Gammaproteobacteria;Alteromonadales;[Chromatiaceae];Rheinheimera |  |  |  | 17% |  |  |  |  |  |  |  |  |  |  |  |  |  |
| Proteobacteria;Gammaproteobacteria;Enterobacteriales;Enterobacteriaceae; |  |  |  | 21% |  |  |  |  |  |  |  |  |  |  |  |  |  |
| Proteobacteria;Gammaproteobacteria;Pseudomonadales;Pseudomonadaceae;Pseudomonas |  |  |  | 10% |  |  |  |  |  |  |  |  |  |  |  |  | 1% |
| Proteobacteria;Deltaproteobacteria;Desulfuromonadales;Geobacteraceae;Geobacter |  |  |  |  |  | 37% | 14% | 22% | 31% | 9% | 19% |  | 34% |  | 19% | 25% |  |
| Proteobacteria;Epsilonproteobacteria;Campylobacterales;Helicobacteraceae;Sulfuricurvum |  |  |  |  |  |  |  |  |  |  |  |  |  |  | 15% |  |  |
